# Pregnancy in Lysinuric Protein Intolerance Complicated by Immune Dysregulation and Severe Thrombocytopenia

**DOI:** 10.1002/jmd2.70109

**Published:** 2026-07-03

**Authors:** Eamon P. McCarron, Melanie Hill, Adam Lomas, Victoria Elliott, Emma Walkinshaw, Tessa Bonnett, Kar‐Ping Kuet, Rachel Tattersall, Claire Mapplebeck

**Affiliations:** ^1^ Adult Inherited Metabolic Disorders, Sheffield Teaching Hospitals NHS Foundation Trust Sheffield UK; ^2^ Faculty of Health, University of Sheffield Sheffield UK; ^3^ The Yorkshire & Humber Maternal Medicine Network Sheffield UK; ^4^ Endocrinology Department Northern General Hospital, Sheffield Teaching Hospitals NHS Foundation Trust Sheffield UK; ^5^ Obstetrics and Maternal Medicine, Jessop Wing, Sheffield Teaching Hospitals NHS Foundation Trust Sheffield UK; ^6^ Rheumatology Department Royal Hallamshire Hospital, Sheffield Teaching Hospitals NHS Foundation Trust Sheffield UK; ^7^ Haematology Department Royal Hallamshire Hospital, Sheffield Teaching Hospitals NHS Foundation Trust Sheffield UK

**Keywords:** hyperammonaemia, immune dysregulation, lysinuric protein intolerance, pregnancy, thrombocytopenia

## Abstract

Lysinuric protein intolerance (LPI) is a rare disorder of dibasic amino acid transport associated with secondary urea cycle defects and immune dysregulation. Pregnancy in LPI is seldom reported and presents significant management challenges. We report a 25‐year‐old woman with genetically confirmed LPI complicated by prior hemophagocytic lymphohistiocytosis (HLH) and systemic lupus erythematosus (SLE) who presented with an unplanned pregnancy. Early gestation was characterised by metabolic instability, including transient hyperammonaemia and elevated orotic acid, which improved with optimisation of nitrogen scavenger therapy and nutritional support. Progressive severe thrombocytopenia and anaemia developed during the second trimester and were managed as presumed immune thrombocytopenia with corticosteroids and intravenous immunoglobulin. Delivery by caesarean section at 35 weeks resulted in favourable maternal and neonatal outcomes. To our knowledge, this represents the first reported case of pregnancy in LPI complicated by established immune dysregulation (HLH and SLE) and severe thrombocytopenia, defining a previously uncharacterised high‐risk phenotype with a favourable outcome.

## Highlights

- First reported pregnancy in lysinuric protein intolerance with established HLH, SLE and severe thrombocytopenia.
- Hyperammonaemia occurred during pregnancy and after antenatal intravenous corticosteroid administration.
- Severe thrombocytopenia required immune‐directed therapy during pregnancy.
- Specialist metabolic and obstetric care achieved favourable maternal and neonatal outcomes.

## 1 Background

Lysinuric protein intolerance (LPI, OMIM #222700) is a rare autosomal recessive disorder of dibasic amino acid transport caused by pathogenic variants in SLC7A7, encoding the y + L amino acid transporter 1 (y + LAT1). Impaired transport of lysine, arginine and ornithine across the basolateral membrane of intestinal and renal epithelial cells results in secondary urea cycle dysfunction and disordered nitrogen metabolism [1].

Clinically, LPI is a multisystem disorder characterised by protein aversion, failure to thrive and hyperammonaemia, with additional complications including pulmonary disease, renal involvement and osteoporosis [2]. Immune dysregulation is increasingly recognised, with associations including HLH, autoimmune disease and cytopenias [3]. Metabolic decompensation may occur during catabolic stress, including infection, fasting or increased physiological demand. Management is centred on protein restriction, citrulline supplementation and nitrogen scavenger therapy [4].

Pregnancy represents a significant metabolic and immunological challenge in LPI, driven by increasing protein requirements and the metabolic demands of rapid foetal growth in late gestation. Published experience remains limited, particularly in the context of co‐existing immune dysregulation. We report the multidisciplinary management of pregnancy in a woman with LPI complicated by hyperammonaemia, immune dysregulation and severe thrombocytopenia.

## 2 Case Description

A 25‐year‐old woman, born to consanguineous parents and the second of two affected siblings, had genetically confirmed LPI due to a homozygous pathogenic SLC7A7 frameshift variant (c.1262del; p.(Pro421ArgfsTer98)) and a history of a prior first‐trimester miscarriage. Her affected sibling had experienced a substantially milder clinical course, illustrating the marked phenotypic variability observed in LPI. The patient's clinical course was characterised by significant immune dysregulation, including two infection‐triggered episodes of HLH at ages 14 and 16 years, and subsequent development of systemic lupus erythematosus (SLE) with joint involvement and lupus nephritis at age 15 years.

Baseline renal function was preserved (eGFR > 90 mL/min/1.73 m^2^), with persistent proteinuria (urine protein–creatinine ratio 100 mg/mmol) and no history of hypertension. Additional comorbidities included hypothyroidism requiring levothyroxine and markedly reduced bone mineral density (lumbar spine *Z*‐score −5.0; total hip *Z*‐score −3.4). At diagnosis, this was associated with profound vitamin D deficiency (< 10 nmol/L), hypophosphataemia (0.56 mmol/L), low serum calcium (2.06 mmol/L) and elevated alkaline phosphatase (282 U/L), a biochemical profile consistent with osteomalacia secondary to severe vitamin D deficiency. There was no history of respiratory involvement, and chest radiographs performed prior to pregnancy showed no evidence of pulmonary disease. Baseline abdominal ultrasound demonstrated no splenomegaly. She also had micronutrient deficiencies, including vitamin D, selenium and folate, which were managed with supplementation, including intramuscular ergocalciferol 300 000 IU every 3 months.

At last review, her weight was 47.1 kg (BMI 20.9 kg/m^2^). Baseline treatment included L‐citrulline (94 mg/kg/day) and sodium benzoate (75 mg/kg/day) in four divided doses. She had not experienced hyperammonaemia since childhood but reported poor adherence to therapy. Nutritional management was complicated by longstanding protein aversion, with an intake of approximately 30 g/day (0.56 g/kg/day), below recommendations [5]. Plasma amino acids were consistent with LPI (Table 1), with normal ammonia and urine orotic acid.

**TABLE 1:** Plasma amino acid profile across pregnancy.

	Reference range	Baseline	1st trimester	2nd trimester	3rd trimester
Taurine	29–211	56	23	12	15
Aspartate	5–52	45	< 5	< 5	7
Threonine	48–195	119	80	60	80
Serine	66–231	160	83	78	86
Glutamic acid	20–180	88	19	36	42
Glutamine	279–695	791	899	721	695
Proline	95–429	520	254	245	292
Glycine	133–455	561	266	200	214
Alanine	182–606	626	427	467	486
Citrulline	10–51	57	56	23	26
Valine	115–339	207	122	94	105
Methionine	11–40	33	21	21	20
Isoleucine	29–102	66	38	35	21
Leucine	62–209	141	60	63	66
Tyrosine	34–127	47	58	34	32
Phenylalanine	35–105	110	43	33	30
Histidine	47–108	75	50	34	48
Ornithine	24–139	53	7	5	< 5
Lysine	73–250	99	49	47	53
Arginine	17–149	77	13	11	7

*Note:* Persistently reduced dibasic amino acids (lysine, arginine and ornithine) throughout pregnancy were consistent with the underlying transport defect. Glutamine remained elevated across gestation, reflecting ongoing impairment of nitrogen handling, while early increases in glycine, alanine and proline improved following optimisation of metabolic and nutritional management. Declining essential amino acid concentrations in later pregnancy likely reflected increased maternal and foetal utilisation, physiological haemodilution and relative protein insufficiency.

### 2.1 First Trimester (0–13 Weeks)

The patient presented at 13 weeks' gestation with an unplanned pregnancy, without prior preconception counselling. Her weight was 48.5 kg (BMI 21 kg/m^2^). Plasma glutamine was elevated (899 μmol/L), urine orotic acid was increased (urine orotic acid–creatinine ratio 27.4 μmol/mmol; reference range 0.0–3.4), and ammonia rose transiently to 91 μmol/L. This normalised following optimisation of metabolic therapy: sodium benzoate was increased to 100 mg/kg/day and administered in two divided doses to improve adherence, while citrulline was continued at the same total daily dose but changed to twice‐daily administration also. Hydroxychloroquine 200 mg daily was continued throughout pregnancy without dose adjustment.

**FIGURE 1:**
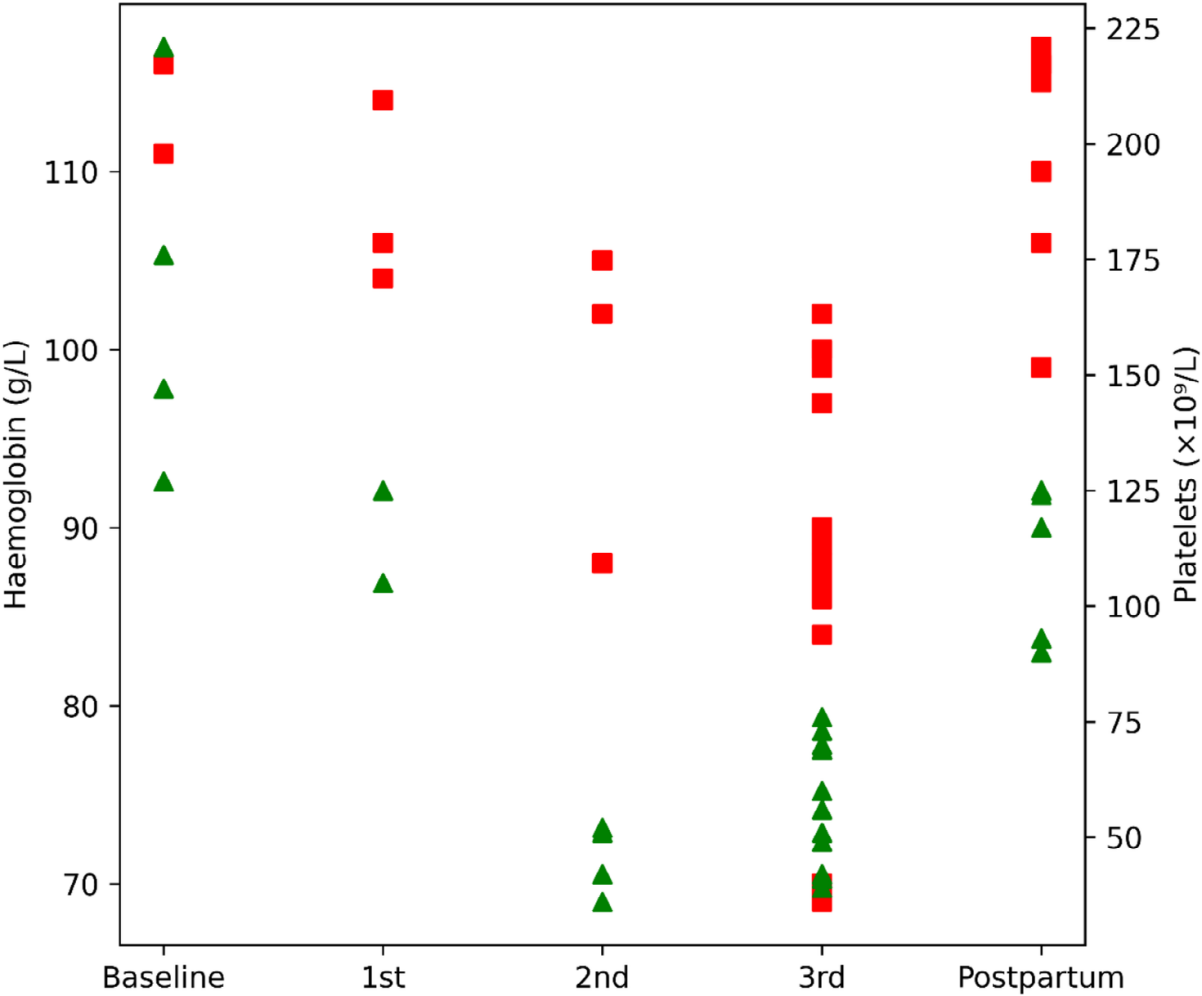
Longitudinal haemoglobin and platelet trends across pregnancy and postpartum. Serial measurements of haemoglobin (g/L; red squares) and platelet count (×10^9^/L; green triangles) from baseline through the first, second, and third trimesters to the postpartum period. Both haemoglobin and platelet counts declined during pregnancy, reaching their lowest values in the third trimester (36 × 10^9^/L). Haemoglobin decreased to 70 g/L and was managed with blood transfusion. Progressive thrombocytopenia was treated with corticosteroids (prednisolone initially 10 mg daily, increased gradually to 20 mg once daily) and intravenous immunoglobulin (1 g/kg administered on two occasions, 2 weeks apart) initiated in the third trimester, with subsequent postpartum recovery of both. Anaemia in pregnancy is typically defined as haemoglobin <110 g/L in the first trimester, < 105 g/L in the second trimester, and < 110 g/L in the third trimester. The lower threshold in the second trimester reflects physiological haemodilution due to disproportionate expansion of maternal plasma volume relative to red cell mass. Gestational thrombocytopenia is usually mild, with platelet counts generally remaining >100 × 10^9^/L. Observed haemoglobin and platelet values remained below expected pregnancy‐specific reference ranges throughout much of gestation.

Thyroid function demonstrated elevated TSH (20 mIU/L) with normal free thyroxine, and levothyroxine dose was increased. Haemoglobin was 114 g/L and platelet count 221 × 10^9^/L at presentation, declining to 105 × 10^9^/L (Figure 1), indicating early thrombocytopenia. Obstetric assessment, including a 12‐week scan, was normal.

Protein requirements during pregnancy were estimated at 40 g/day [6], although the patient tolerated up to 50 g/day by the end of the first trimester with additional high‐calorie supplementation. Fat emulsion supplementation (Fresubin 5 kcal/mL) was introduced as an alternative to glucose polymers, which were poorly tolerated (Table 2). Pregnancy multivitamin and mineral supplementation containing folic acid, vitamin D, iodine and calcium was also initiated.

**TABLE 2:** Nutritional management and dietary targets across pregnancy and postpartum in lysinuric protein intolerance.

	1st trimester	2nd trimester	3rd trimester	Postpartum
Weight (kg)	48.5	49.9	55.1	52
Protein target (g/day)	41	50	70	25 restriction for first 5 days then gradual increase to 50
Calorie target (Kcal/day)	1950	2237	2416	2200
Supplement	Fat emulsion	Fat emulsion	Fat emulsion and glucose polymer	Glucose polymer

*Note:* Nutritional management was adjusted throughout pregnancy and the postpartum period according to metabolic stability, gestational requirements, and treatment tolerance.

### 2.2 Second Trimester (14–27 Weeks)

The patient was managed jointly in an inherited metabolic disease and maternal medicine clinic along with haematology and rheumatology. Weight increased to 49.9 kg (BMI 21.6 kg/m^2^). Metabolic control improved, with ammonia remaining within the normal range and urine orotic acid normalising. Protein intake increased to approximately 60 g/day, supported by continued fat emulsion supplementation to maintain caloric intake. Essential amino acid supplementation was not tolerated.

The clinical course was complicated by progressive severe thrombocytopenia, with platelet counts declining from 221 × 10^9^/L to a low of 36 × 10^9^/L (Figure 1). Haemoglobin ranged from 87 to 105 g/L. The degree of thrombocytopenia was substantially greater than expected for gestational thrombocytopenia, while the reduction in haemoglobin exceeded that anticipated from physiological haemodilution alone. Blood film confirmed thrombocytopenia with features of mixed anaemia (mean cell volume (MCV) between 90 and 100 fL). Haptoglobin was low (0.17 g/L) without red cell fragmentation on blood film. Liver function tests remained normal. Iron deficiency was treated with intravenous iron and B_12_ deficiency intramuscular hydroxocobalamin. Prothrombin, APTT and fibrinogen remained normal throughout pregnancy. Although ferritin was 148 μg/L prior to treatment, interpretation was limited by chronic inflammatory disease and pregnancy, and intravenous iron was administered on haematology advice as part of the management of multifactorial anaemia. Autoimmune markers showed borderline low C3 (0.83 g/L), normal C4 (0.16 g/L) and negative anti–double‐stranded DNA antibodies, with normal CRP and no biochemical evidence of active SLE. Urine protein–creatinine ratio increased from 100 mg/mmol at baseline to 144 mg/mmol during pregnancy, indicating progressive proteinuria despite preserved blood pressure and eGFR (> 90 mL/min/1.73 m^2^). Foetal growth remained appropriate, although polyhydramnios was noted at 28 weeks and foetal weight was at the 25th centile.

At 27 weeks' gestation, the patient was admitted with lower limb swelling; deep vein thrombosis was excluded. Ferritin was elevated at 1319 μg/L following intravenous iron therapy. Inflammatory markers were not raised, triglycerides were normal (1.4 mmol/L), fibrinogen 2.0 g/L and there was no pyrexia or rash. An Hscore [7] of 72 indicated a low probability of HLH. She was managed conservatively with close monitoring and encouraged to monitor her temperature at home.

### 2.3 Third Trimester (28+ Weeks) and Delivery

Weight increased to 55.1 kg (BMI 23.9 kg/m^2^), representing a total gestational weight gain of 7.6 kg, below the recommended range of approximately 11.5–16 kg [8]. This likely reflected intermittent adherence to dietary recommendations and nutritional supplementation. Thrombocytopenia progressed and, in the absence of biochemical evidence of lupus flare or coagulopathy, was managed as presumed immune thrombocytopenia. Complement testing demonstrated reduced C4 (0.09 g/L) with preserved C3 (0.98 g/L), and anti–double‐stranded DNA antibodies remained normal. Prednisolone (initially 10 mg daily, increased gradually to 20 mg once daily) and intravenous immunoglobulin (1 g/kg administered on two occasions, 2 weeks apart) were initiated, with partial haematological response. She developed steroid‐induced diabetes which was monitored (managed conservatively without modification of the metabolic diet). As thrombocytopenia worsened, prophylactic low‐molecular‐weight heparin was discontinued when the platelet count fell below 60 × 10^9^/L owing to concerns regarding bleeding risk. Protein intake was increased to approximately 70 g/day.

Renal involvement progressed, with worsening proteinuria and mild haematuria, while blood pressure and eGFR remained preserved. The patient was admitted with per vaginal bleeding, and elective caesarean section was planned. During admission, hyperammonaemia occurred (peak 171 μmol/L) within 24 h of intravenous antenatal corticosteroid administration for foetal lung maturation. Management included intravenous dextrose (2 mL/kg/h) and intravenous nitrogen scavenger therapy with sodium phenylbutyrate (250 mg/kg/day) with subsequent improvement.

Caesarean section was performed at 35 weeks. Estimated blood loss was approximately 800 mL, with postpartum haemoglobin of 70 g/L requiring transfusion. The patient received intravenous dextrose (2 mL/kg/h) throughout delivery and for 72 h postpartum.

### 2.4 Postpartum

A live infant weighing approximately 2 kg was delivered and required short‐term admission to the special care baby unit before discharge in good condition. The partner was consanguineous; however, neonatal ammonia and plasma amino acids were normal. Breastfeeding was initially deferred, with colostrum harvesting encouraged; however, the patient wished to breastfeed and was supported with advice regarding adequate caloric intake. Feeding was established with combined breastfeeding, formula supplementation, and calcium supplementation, although breastfeeding was discontinued by week 3 postpartum. At 4 weeks postpartum, the patient sustained acute T10/L1 vertebral fragility fractures following a fall; this was managed with analgesia and specialist metabolic bone follow‐up. In the postpartum period, maternal platelet counts normalised, ammonia remained stable, and prednisolone was successfully weaned. Urine protein–creatinine ratio decreased to 60 mg/mmol post‐partum.

## 3 Discussion

This case highlights pregnancy in LPI as a complex clinical state defined by the interaction between metabolic vulnerability and immune dysregulation. It is particularly notable due to established immune pathology, including prior HLH and SLE, a combination rarely described in pregnancy. Cytopenias in LPI are well recognised and likely multifactorial, reflecting bone marrow involvement, immune‐mediated destruction and physiological adaptations of pregnancy [9]. While immune abnormalities are well described, most reported pregnancies involve patients without significant baseline autoimmune disease, and thrombocytopenia is typically mild (or occasionally moderate) [10, 11, 12, 13]. This case therefore represents a distinct high‐risk phenotype in which pre‐existing immune dysfunction amplifies both diagnostic complexity and susceptibility to decompensation. The presence of low birth weight is consistent with previously reported foetal growth complications and underscores the importance of early monitoring and awareness [10, 11]. Cohort data from Finland, where there is a known founder mutation, further demonstrate an increased risk of anaemia, thrombocytopenia, bleeding and foetal growth restriction [10, 14].

Favourable outcomes in reported cases depend on close metabolic and obstetric monitoring [11, 12]. In this case, progressive severe thrombocytopenia during the second and third trimesters, in the absence of biochemical SLE activity or coagulopathy, was most consistent with immune‐mediated thrombocytopenia. Literature describing haemophagocytic features and macrophage activation in LPI supports an immune‐driven mechanism, indicating that cytopenias may reflect underlying immune dysregulation rather than primary obstetric pathology [3]. Although thrombocytopenia occurs in up to 10% of pregnancies, severe or progressive decline necessitates evaluation for pre‐eclampsia, HELLP syndrome and thrombotic microangiopathy [15]. In LPI, baseline cytopenias, renal involvement and hepatic abnormalities may mimic these conditions; however, preserved blood pressure and absence of haemolysis or coagulopathy in this case made obstetric microangiopathy unlikely despite progressive proteinuria. Treatment with corticosteroids and intravenous immunoglobulin resulted in a partial haematological response and was consistent with standard management of immune thrombocytopenia in pregnancy [15]. The anaemia was normocytic (MCV 90–100 fL) and multifactorial, reflecting physiological haemodilution, chronic immune‐mediated suppression of erythropoiesis, and relative protein and amino acid insufficiency; vitamin B_12_ deficiency was excluded following replacement.

Metabolic vulnerability was a central feature. Hyperammonaemia occurred both in early gestation and within 24 h of antenatal corticosteroid administration, highlighting susceptibility to catabolic stress. Pregnancy and the postpartum period are recognised high‐risk states for hyperammonaemia in urea cycle disorders, even in previously stable individuals, with nitrogen scavenger therapies forming a key component of management [16]. Corticosteroids may contribute to hyperammonaemia through suppression of urea cycle enzyme expression and increased ammonia production [17]. In this case, low‐dose oral corticosteroids were tolerated, whereas hyperammonaemia developed within 24 h of antenatal intravenous corticosteroid administration, suggesting a potential association and underscoring the need for anticipatory metabolic support when high‐dose intravenous corticosteroids are required.

Dietary adherence during pregnancy can be challenging in patients with LPI and other inherited metabolic disorders [18, 19]. Poor compliance with prescribed supplements in this case necessitated the use of food fortification strategies to support nutritional intake. The patient intermittently tolerated higher protein intake than prescribed during the first and second trimesters, although intake remained variable. Overall, approximately 90% of estimated energy and protein requirements were achieved by the end of pregnancy, which may have contributed to suboptimal gestational weight gain.

Renal involvement progressed, with increasing proteinuria despite preserved glomerular filtration rate, consistent with recognised renal manifestations of LPI [10]. Postpartum recovery was favourable, with resolution of thrombocytopenia and stabilisation of ammonia and haemoglobin. However, the postpartum period remains a critical window, with risk of both metabolic decompensation and immune activation, including HLH, necessitating continued surveillance and use of validated scoring systems where appropriate [7, 20].

Breastfeeding has been reported in LPI and can be supported with multidisciplinary input [10]. Lactation represents a period of increased metabolic demand, requiring augmented caloric intake and close biochemical monitoring. In addition, the postpartum period is also recognised as a time of increased metabolic risk due to uterine involution and the abrupt cessation of fetoplacental nitrogen utilisation; however, plasma ammonia remained within the normal range throughout the postpartum period in our patient. In our case, breastfeeding was initially discouraged and then cautiously initiated (due to patient preference) with colostrum harvesting and ammonia monitoring and subsequently established in combination with formula feeding. The patient had markedly reduced bone mineral density at baseline and was counselled regarding the potential impact of lactation on calcium balance and fracture risk. The markedly reduced bone mineral density in this case was considered multifactorial but likely reflected chronic osteomalacia related to profound vitamin D deficiency, hypophosphataemia, low calcium levels, nutritional impairment, and the underlying metabolic disorder [21, 22]. Pregnancy and lactation may have further increased calcium demands and contributed to fracture risk in a patient with pre‐existing severe metabolic bone disease. Although successful treatment of osteoporosis with alendronate has been reported in LPI [23], the biochemical profile at diagnosis suggested impaired bone mineralisation as a major contributor to the low bone mineral density in this case. Consequently, management prioritised correction of nutritional deficiencies and optimisation of bone mineralisation through calcium supplementation and continued high‐dose intramuscular ergocalciferol replacement, with specialist metabolic bone follow‐up. Despite these measures, she remained at high baseline risk of fragility fracture and subsequently sustained acute vertebral fractures postpartum following a fall.

## 4 Conclusion

Despite these challenges, a favourable maternal and foetal outcome is achievable in LPI complicated by significant baseline autoimmune disease through coordinated multidisciplinary management, proactive metabolic optimisation and careful anticipation of haematological and obstetric complications. To our knowledge, this represents the first reported case of pregnancy in LPI complicated by established immune dysregulation (HLH and SLE) and severe thrombocytopenia, defining a previously uncharacterised high‐risk phenotype.

## Funding

The authors have nothing to report.

## Conflicts of Interest

The authors declare no conflicts of interest.

## Data Availability

The data that support the findings of this study are available on request from the corresponding author. The data are not publicly available due to privacy or ethical restrictions.
